# Targeting Ovarian Cancer Cell Cytotoxic Drug Resistance Phenotype with *Xanthium strumarium* L. Extract

**DOI:** 10.1155/2019/6073019

**Published:** 2019-11-15

**Authors:** Marbelis Francisco Fernandez, Cyndia Charfi, Janet Piloto-Ferrer, Maria Lidia González, Sylvie Lamy, Borhane Annabi

**Affiliations:** ^1^Laboratoire d'Oncologie Moléculaire, Département de Chimie, Centre de Recherche BIOMED, Université du Québec à Montréal, Montreal, Quebec, Canada; ^2^Departamento de Genética Toxicológica, Centro de Investigación y Desarrollo de Medicamentos (CIDEM), Avenida 26, No. 1605 e/Puentes Grandes y Boyeros, La Habana, Cuba

## Abstract

Emerging drugs aim at targeting the genomic integrity and replication machinery in ovarian cancer. While the antiproliferative activity of *Xanthium strumarium* L. extract (XFC), a traditional herbal medicine, is believed to alter the mitotic apparatus of Chinese hamster ovary epithelial cells, its capacity to target and overcome the chemoresistance phenotype in ovarian cancer is unknown. Among the cancer cell lines tested, we found that the best proliferation inhibitory effect for XFC was against ovarian cancer cells and ranged from 30 to 35 *μ*g/mL. XFC efficiently targeted both the cytotoxic drug chemoresistance phenotype of SKOV-3 cells and of the chemosensitive ES-2 cells. Early apoptosis and late apoptosis were effectively induced by XFC extract in ES-2 cells, whereas late apoptosis and necrosis events were triggered in SKOV-3 cells. Cell cycling regulation was trapped by XFC extract in the G2/M phase in both the ES-2 and SKOV-3 cell models. This effect was, in part, attributable to increased dose-dependent tubulin polymerization, which was increased in SKOV-3 cells. Whereas XFC extract triggered poly (ADP-Ribose) polymerase (PARP) cleavage in both ES-2 and SKOV-3 cells, it only lowered Nrf2 in ES-2 cells and phosphorylated Akt levels in SKOV-3 cells. Interestingly, cell cycling regulators Cdk4, Cyclin D3, and p27 were all decreased in SKOV-3 cells. XFC extracts were effective in inhibiting *in vitro* migration in both ovarian cancer cell models. Our data support the potential anticancer targeting of chemoresistant human ovarian cancer cells phenotype by XFC extract.

## 1. Introduction

Ovarian cancer is the leading cause of gynecological cancer mortality. Despite the fact that first-line chemotherapy is effective in reducing tumor burden following cytoreductive surgery, the 5-year survival rate for stage III and IV disease is ∼20–30% [1]. One of the major reasons for this low survival rate is the onset of drug resistance. Attempts to overcome this resistance to antitumor drugs in ovarian cancer have resulted in the combination chemotherapy of cisplatin (CDDP) and Taxol as the first-line therapeutic protocol via long-term prospective studies of clinical trials [2]. Although many tumor cells in humans gradually acquired resistance during chemotherapy, our understanding of drug resistance mechanisms remains insufficient to overcome clinical failure. Differential molecular and cellular studies using chemoresistant and chemosensitive cell line models can thus serve as an initial screen for agents that can circumvent drug resistance phenotypes.

Resistant cell lines, selected by exposure to antitumor agents, have been valuable tools for the identification of the factors underlying *in vitro* drug resistance. The use of these resistant cell lines has greatly enhanced our understanding of the mechanisms of resistance and of drug resistance-associated genes, such as multidrug resistance gene 1 (MDR1) and glutathione S-transferase pi (GST-pi) [3, 4]. However, a crucial problem is that studies with cells in culture may not always reflect the situation in clinical tumors and contradictory evidence concerning the mechanisms of drug resistance has been reported [5–9]. This situation may be due, at least in part, to differences between the resistant cell lines selected by different approaches and a failure in combination of the laboratory and the clinic.

Throughout history, plants have been the main sources in the discovery of natural-based medicines. In the anticancer area, plant-derived agents such as the Vinca alkaloids, the epipodophyllotoxins, the taxanes, and the camptothecin derivatives are among the most effective cancer chemotherapeutics currently available [10]. The search for new phytochemicals for cancer therapy is therefore a worthwhile endeavor, and the identification of anticancer plant compounds is usually begun by collecting a variety of samples from around the world or by relying on folklore. This is the case for the plant *Xanthium strumarium* L. (Family: Asteraceae) which exhibits a global distribution and is found abundantly throughout Eurasia and America [11]. Historically, *Xanthium* species have been used as traditional herbal medicines in oriental countries. They have also been used abundantly as analgesics, as antibacterial and anti-inflammatory agents, and have been used for chronic bronchitis, chronic rhinitis, and allergic rhinitis as well as to relieve constipation, diarrhea, and vomiting [11]. Moreover, plant infusions have been used in the treatment of rheumatism and kidney diseases [12]. It has also been reported that the genus *Xanthium* is a source for many interesting compounds such as sesquiterpene lactones with xanthanolide-type skeletons that have significant antitumor activity in a variety of cell culture systems [13–16], with terpenoids, thiazolidinediones, sterols, and caffeoylquinic acid as major secondary metabolites. Despite the many studies carried out on *Xanthium strumarium* L., the cellular and molecular mechanisms underlying the anticancer actions of this plant remain poorly characterized.

In the present study, we induced apoptosis in SKOV-3 cells, an established human epithelial ovarian cancer cell line model resistant both to tumor necrosis factor and to several cytotoxic drugs including diphtheria toxin, cis-platinum, and Adriamycin [8], and compared these with results with established chemosensitive ES-2 ovarian cancer cells. We investigated the effects of *Xanthium strumarium* L. extract (XFC) administration and assessed its potential to circumvent the drug resistance phenotype in the SKOV-3 chemoresistant ovarian cancer cell model. We provide herein evidence suggesting that the XFC content in anticancer molecules could efficiently target and circumvent the molecular processes that contribute to ovarian cancer cell resistance to current cytotoxic therapies.

## 2. Materials and Methods

### 2.1. Materials

Sodium dodecyl sulfate (SDS) and bovine serum albumin (BSA) were purchased from Sigma (Oakville, ON). Cell culture media were obtained from Life Technologies (Burlington, ON). Electrophoresis reagents were purchased from Bio-Rad (Mississauga, ON). The enhanced chemiluminescence (ECL) reagents were from Amersham Pharmacia Biotech (Baie d'Urfé, QC). Micro bicinchoninic acid protein assay reagents were from Pierce (Rockford, IL). The monoclonal antibody against GAPDH (glyceraldehyde 3-phosphate dehydrogenase) was from Advanced Immunochemical Inc. (Long Beach, CA). The Alexa Fluor® 488 donkey anti-rabbit IgG antibody was purchased from Invitrogen (Carlsbad, NM). Polyclonal antibodies against Survivin, Nrf2, AKT, and phospho-AKT, PARP, Cyclin D1, Cyclin D3, Cdk2, Cdk4, Cdk6, and monoclonal antibody against p27 were from Cell Signaling Technology (Beverly, MA).

### 2.2. Plant Material and Preparation of Chloroform Extract of *Xanthium strumarium* L. (XFC)

*Xanthium strumarium* L. was collected from the Medicinal Plants Experimental Station “Dr. Juan Tomás Roig” in San Antonio de los Baños city (Artemisa, Cuba). A voucher specimen with number ROIG 4594 was deposited at the herbarium of this institution. Plant parts (500 g) were extracted with 70% ethanol as described elsewhere [17]. A fluid extract was prepared from the dried material by hydroalcoholic extraction in using four rounds of percolation. It was concentrated under reduced pressure to obtain a raw extract (whole extract). Extraction of chloroform-soluble compounds was described in detail previously [18]. Chloroform was removed by reduced pressure (Büchi Rotavapor), and the residual pale yellow oil was resuspended in dimethyl sulfoxide (DMSO) for the biological evaluations. DMSO (1% final concentration) served as a negative control condition in all untreated cell conditions. This *Xanthium strumarium* L. chloroform extract (XFC extract) was used throughout the study to treat cells.

### 2.3. Breast, Ovarian, and Colorectal Cancer Cell Cultures

Human SKOV-3 ovarian adenocarcinoma cells as well as human ES-2 ovarian clear cell carcinoma cells were purchased from the American Type Culture Collection (ATCC). Cells were grown as a monolayer with McCoy's 5a Medium Modified (Wisent, 317-010-CL) containing 10% fetal bovine serum (Life Technologies, 12483-020), 100 U/mL penicillin, and 100 mg/mL streptomycin (Wisent, 450-202-EL). All other cell lines used in the study were from ATCC. MDA-MB-231 and MCF-7 (breast cancer cells) as well as HT-29 and C2BBe-1 (colon cancer cells) were grown in Eagle's Minimum Essential Medium (Wisent, 320-036-CL) or in Dulbecco's Modified Eagle's Medium (319-005-CL), respectively, containing 10% FBS and antibiotics. All the cells were cultured at 37°C under a humidified 95%–5% (v/v) mixture of air and CO_2_.

### 2.4. Impact of XFC on Cancer Cell Proliferation

The MDA-MB-231, MCF-7, ES-2, SKOV-3, HT-29, and C2BBe1 cells were seeded in complete medium (10^3^ cells/200 *μ*L/well) in 96-well plates, allowed to adhere for 6 hours, and the medium was supplemented with increasing XFC concentrations ranging from 1 to 1000 *μ*g/mL (200 *μ*L/well, final volume). Cell proliferation was measured after 72 hours using the MTT [3-(4,5-dimethylthiazol-2-yl)-2,5-diphenyltetrazolium bromide] cytotoxicity assay in accordance with the protocol described by Mosmann with the following modifications [19]. The cells were incubated with MTT (0.5 mg/mL) at 37°C under a humidified atmosphere containing 5% CO_2_ for 4 hours. After incubation, 100 *μ*L DMSO (solubilizing reagent) was added to each well and mixed thoroughly to dissolve the dark blue crystals. The presence of viable cells was visualized by the development of a purple color due to the formation of formazan crystals. The plates were read on a SpectraMax Plus reader (Molecular Devices, Sunnyvale, CA) using a test wavelength of 570 nm and reference wavelength of 630 nm. The IC_50_ values of the samples were determined from a dose–response curve obtained by using seven different sample concentrations (6.25, 12.5, 25, 50, 100, 200, and 400 *μ*g/mL). Analyses were made in triplicate for each condition.

### 2.5. Apoptosis Analysis

Apoptosis was determined by FITC-labeled Annexin V/PI double staining and flow cytometry analysis. Briefly, SKOV-3 and ES-2 cells were treated with XFC at 12.5, 25, 50, 100, 200, and 400 *μ*g/mL for 24 or 48 hours. At the indicated time, cells were harvested and fixed, and apoptosis was then measured using the FITC Annexin V Apoptosis Detection Kit II (BD Biosciences, Mississauga, ON) according to the manufacturer's protocol. The percentages of cells within early and late stages of apoptosis were determined using a BD Accuri C6 flow cytometer. The data were analyzed using FlowJo 10.1 software. At least 10,000 cells were counted for each measurement. The following controls were used to set up gates: unstained cells, cells with FITC Annexin V only, and cells with PI only.

### 2.6. Cell Cycle Analysis

ES-2 and SKOV-3 cells were incubated for 48 hours in medium without FBS at 37°C in 5% CO_2_ for synchronization of the cell cycle. Cells were then treated for 24 hours with either 0.1% DMSO (controls) or various concentrations of XFC (20, 30, 40, 50, or 60 *μ*g/mL). Treatments were performed in medium containing 10% FBS. At the end of treatment, the cells were collected by mild trypsin digestion, washed with ice-cold PBS, and fixed in ice-cold 70% ethanol in PBS overnight at −20°C. Cells were then centrifuged at 10,000 rpm for 10 min, followed by careful removal of the supernatant. Three volumes of staining solution in PBS, containing propidium iodide (PI, 40 *μ*g/mL; Calbiochem, San Diego, CA) and DNase-free RNase (100 *μ*g/mL), were added for at least 30 min at 37°C in the dark before analysis. The proportion of the cell population in each phase of the cell cycle was determined as a function of the DNA content using a BD Accuri C6 flow cytometer (BD Biosciences, Mississauga, ON). Cell cycle analysis was performed with the BD AccuriTM C6 software (version 1.0.264.21). For each measurement, at least 10,000 cells were counted.

### 2.7. Immunofluorescence Staining of Tubulin Cytoskeleton

ES-2 and SKOV-3 cells were seeded on cover slips and treated with the indicated concentrations of XFC or with nocodazole (50 ng/mL) for 24 hours. Cells were rinsed in PHEM buffer (60 mM PIPES, 25 mM Hepes, 10 mM EGTA, 2 mM MgCl_2_), fixed in 4% formaldehyde for 15 min at room temperature and permeabilized with 0.5% Triton X-100 for 5 min. After the reaction was blocked in 10% BSA/PBS, the cells were incubated with mouse anti-*α*-tubulin antibody (1/2,000; T5168; Sigma) for 1 hour at room temperature. After several washes, the cells were incubated with Alexa Fluor-488 conjugated anti-rabbit IgG antibody (1/1,000 dilution) for 1 hour at room temperature. The cell nuclei were visualized with 1 *μ*g/mL DAPI staining for 5 min. Slides were then dried and mounted with the ProLong Gold antifade reagent (Fisher Scientific, Ottawa, ON). The cells were visualized with an A1R Nikon confocal unit attached to an inverted Eclipse Ti microscope using a Plan Apo 60/1.4 NA oil objective (Nikon Canada, Mississauga, ON). The resulting micrographs were acquired and processed with NIS Element (Nikon Canada) and ImageJ (U.S. National Institutes of Health, Bethesda, MD) software packages.

### 2.8. Immunoblotting Assessment of PARP Cleavage and of Cell Cycle Regulators

Proteins from control and treated cells were separated by SDS-polyacrylamide gel electrophoresis (PAGE). After electrophoresis, proteins were electrotransferred to polyvinylidene difluoride membranes which were then blocked for 1 hour at room temperature with 5% nonfat dry milk in Tris-buffered saline (150 mM NaCl, 20 mM Tris-HCl, pH 7.5) containing 0.3% Tween-20 (TBST). Membranes were further washed in TBST and incubated with the primary antibodies (1/1,000 dilution) in TBST containing 3% BSA, followed by a 1-hour incubation with horseradish peroxidase-conjugated anti-rabbit IgG (1/2,500 dilution) in TBST containing 5% nonfat dry milk. Immunoreactive material was visualized with the ECL detection system. The immunoreactive bands were quantified using ImageJ software (NIH).

### 2.9. Wound-Healing Assay

ES-2 and SKOV-3 cells were seeded into 6-well tissue culture dishes and grown to nearly confluent cell monolayers. Then, a linear wound was generated in the monolayer with a sterile 200 *μ*L pipette tip creating a cell-free area [20]. Cultures were gently washed with the growth medium to remove loose cells. The cells were then treated with either vehicle or XFC (60 *μ*g/mL) in culture medium supplemented with 1% FBS. Cells for control conditions were also scratched, washed, and maintained in culture medium supplemented with 1% FBS after the scratch. Immediately after the scratch and at 4, 8, and 24 h, at least four images of the scraped area were captured using phase contrast microscopy and analyzed using ImageJ software. Two independent experiments were performed, using three wells for each stimulating condition. The same scratched area was selected for the measurements at each time of the study.

### 2.10. Statistical Data Analysis

Data are representative of three or more independent experiments. Statistical significance was assessed using Student's unpaired *t*-test and was used to compare the XFC effect to vehicle-treated cells. All statistical analyses and graphs were performed using the GraphPad Prism software version 5.0b (San Diego, CA). Where indicated, data were presented as means ± SD of three independent experiments (significance: ^*∗*^*p* < 0.05, ^*∗∗*^*p* < 0.01, ^*∗∗∗*^*p* < 0.001 versus the negative control).

## 3. Results

### 3.1. Impact of the *Xanthium strumarium* L. Extract (XFC) on Breast, Ovarian, and Colorectal Cancer Cells Proliferation

**Figure 1:**
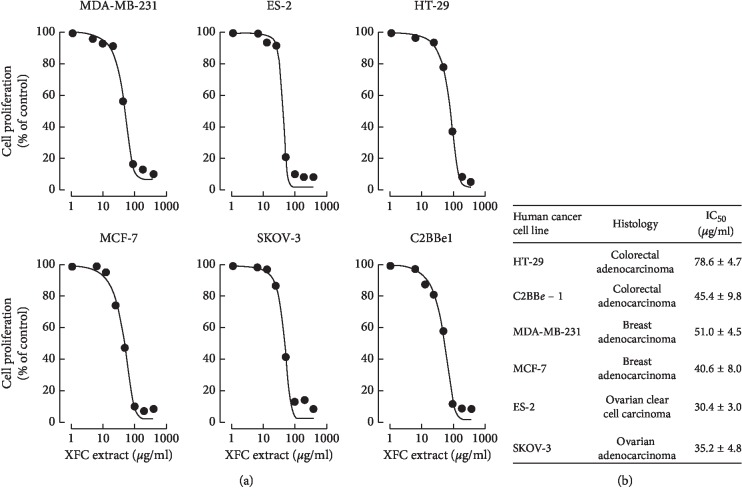
Impact of the *Xanthium strumarium* L. extract (XFC) on breast, ovarian, and colorectal cancer cells proliferation. Mid-log phase human cancer cell models derived from breast adenocarcinomas (MCF-7, MDA-MB-231), colorectal adenocarcinomas (HT-29, C2BBe-1), ovarian clear cell carcinoma (ES-2), and ovarian adenocarcinoma (SKOV-3) were seeded in 96-well plates at 10^3^ cells/well density in 200 *μ*L medium and cultured as described in Section 2. Cells were then (a) treated with various concentrations of XFC for 72 hours and then incubated with MTT (0.5 mg/mL) for 4 hours in order to quantify cell proliferation (a representative curve is shown for each cell line). (b) IC_50_ values were obtained for cell line treatments, which were performed in triplicate.

The antiproliferative capacity of XFC was first assessed on established human cancer cell models derived from breast adenocarcinomas (MCF-7, MDA-MB-231), from colorectal adenocarcinomas (HT-29, C2BBe-1), from ovarian clear cell carcinoma (ES-2), or from ovarian adenocarcinoma (SKOV-3). XFC was able to dose-dependently inhibit cell proliferation (Figure 1(a)), with the greatest inhibition potential observed against the two ovarian cancer cell models where IC_50_ values ranged from 30 to 35 *μ*g/mL (Figure 1(b)). This suggests that XFC possesses the ability to inhibit cancer cell proliferation, with the strongest effect against ovarian cancer cells.

### 3.2. XFC Triggers Late Apoptosis in ES-2 Ovarian Cancer Cells

**Figure 2:**
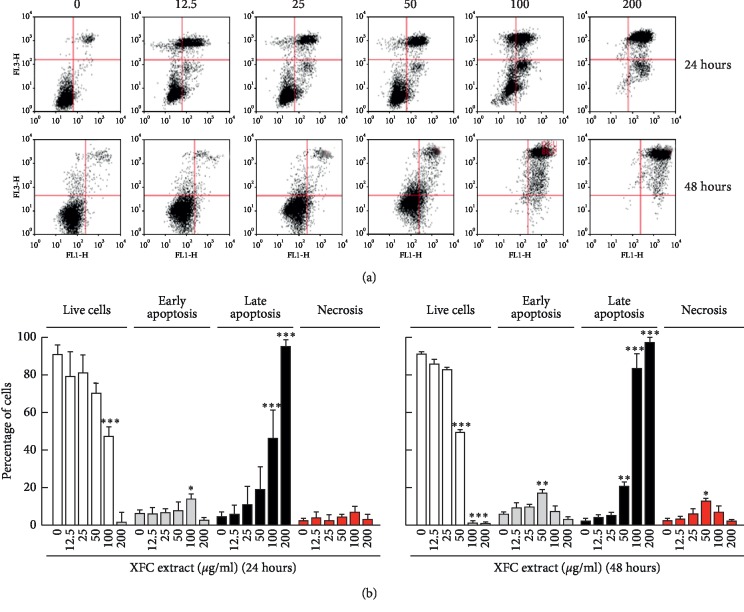
XFC triggers late apoptosis in ES-2 ovarian cancer cells. Human ovarian clear cell carcinoma cells (ES-2) were cultured and treated with increasing concentrations of XFC in serum-free media for 24 or 48 hours, followed by fixation, PI staining, and (a) data acquisition by flow cytometry as described in Section 2 to assess cell death phases. (b) Data analysis was also performed as described in Section 2 in order to assess levels of live cells, early apoptosis, late apoptosis, and necrosis.

**Figure 3:**
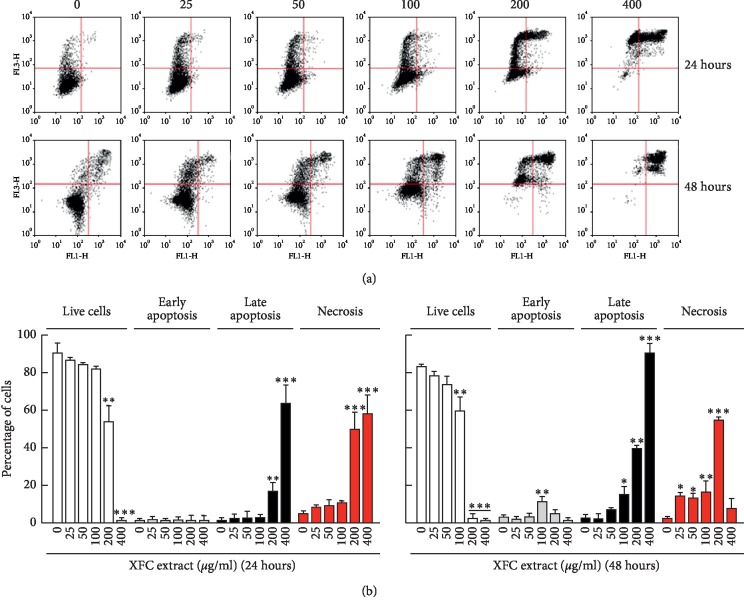
XFC triggers late apoptosis and necrosis in SKOV-3 ovarian cancer cells. Human ovarian adenocarcinoma (SKOV-3) cells were cultured as described in Section 2. Treatment with various concentrations of XFC was performed in serum-free media for 24 or 48 hours, followed by fixation, PI staining, and (a) data acquisition by flow cytometry, as described in Section 2 to assess cell death phases. (b) Data analysis was also performed as described in Section 2 in order to assess levels of live cells, early apoptosis, late apoptosis, and necrosis stages.

Whether XFC triggered any proapoptotic events was next assessed after 24 and 48 hours of XFC treatment by flow cytometry (Figures 2(a) and 3(a)). We found that early apoptosis and late apoptosis were effectively induced by XFC in ES-2 cells and increased with time (Figure 2(b)), whereas late apoptosis and necrosis events were triggered in SKOV-3 cells (Figure 3(b)). XFC can therefore efficiently target the effective cytotoxic drug chemoresistance phenotype reported for SKOV-3 as well as the chemosensitive ES-2 cells.

### 3.3. XFC Alters ES-2 and SKOV-3 Cell Cycle Division

**Figure 4:**
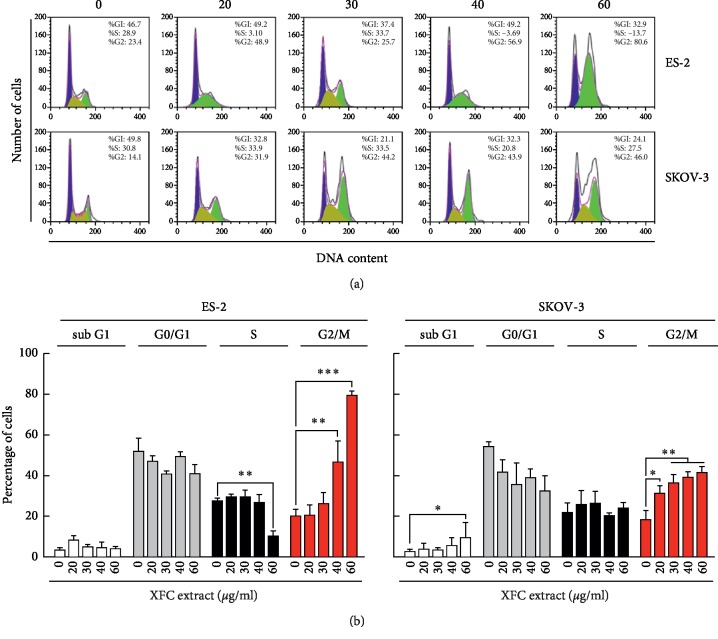
XFC extract alters ES-2 and SKOV-3 cell cycle division. Human ovarian clear cell carcinoma (ES-2) and human ovarian adenocarcinoma (SKOV-3) cells were cultured, followed by treatments with various concentrations of XFC in serum-free media for 24 hours, fixation, and by PI staining. (a) Data acquisition was performed by flow cytometry as described in Section 2 in order to assess cell cycle phases. (b) Data analysis was also performed in order to assess the levels of cells in sub-G1, G0/G1, S, and G2/M phases. Significance: ^*∗*^*p* < 0.05, ^*∗∗*^*p* < 0.01, ^*∗∗∗*^*p* < 0.001 versus the negative control.

Given its antiproliferative and proapoptotic effects, we next addressed how XFC could alter cell cycle division by assessing sub-G1, G0/G1, S, and G2/M phases (Figure 4(a)). Cells were trapped in the G2/M phase upon XFC treatment of both ES-2 and SKOV-3 cell models (Figure 4(b), red bars). This again suggests that XFC can significantly alter molecular events regulating cell division processes controlling cell proliferation.

### 3.4. XFC Alters Tubulin Cytoskeleton in SKOV-3 Ovarian Cancer Cells

**Figure 5:**
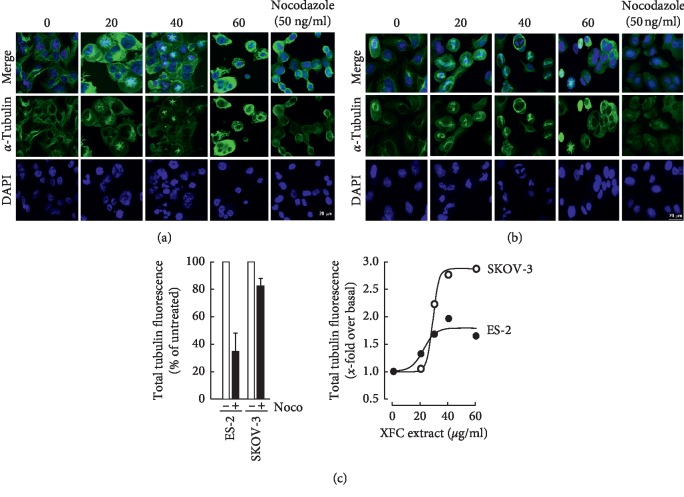
Preferential alteration of the tubulin cytoskeleton in the SKOV-3 ovarian cancer cells by XFC. Human ovarian clear cell carcinoma (ES-2) and human ovarian adenocarcinoma (SKOV-3) cells were cultured as described in Section 2. Treatment with various concentrations of XFC or with nocodazole was performed in serum-free media for 24 hours. Immunostaining of tubulin was performed with antitubulin antibody. DAPI was used as a nuclear stain. Fluorescence microscopy was used for data acquisition in (a) ES-2 and (b) SKOV-3 ovarian cancer cells. (c) Total fluorescence was acquired and the data was processed as described in Section 2.

Increased tubulin levels and decreased polymerization ratio are hallmarks of resistant cells [21]. Here, we tested whether the antimitotic activity of XFC extract altered tubulin polymerization [22] in human ovarian cancer cells, and this was compared to the microtubules depolymerizing agent Nocodazole [23]. We found that XFC altered microtubules organization (Figures 5(a) and 5(b)) as it increased tubulin polymerization in SKOV-3 cells as compared to ES-2 cells (Figure 5(c)). By examining the morphologies of microtubules and chromosomes, we found increased multipolar mitotic spindles consequent to the aggregation of asters as well as increased monopolar spindles consequent to nonseparated centrioles in both cells treated with XFC. In agreement with this, the chromosomes remained condensed but were not able to align properly, which can be explained by the formation of the multipolar acentrosomal spindle leading to a failure of chromosomal segregation. In addition, treated cells that were able to eventually exit mitosis showed aberrations associated with abnormal karyokinesis (increased frequency of cells bearing micronuclei) and failure of cytokinesis (increase in cells containing multiple nuclei) (Figures 5(a) and 5(b)). Such phenomena are typical outcomes of antimitosis drugs. Taken together, these data indicate that XFC directly affects mitosis progression by disrupting the assembly of the normal mitotic spindle.

### 3.5. XFC Signals PARP Cleavage in SKOV-3 Ovarian Cancer Cells

**Figure 6:**
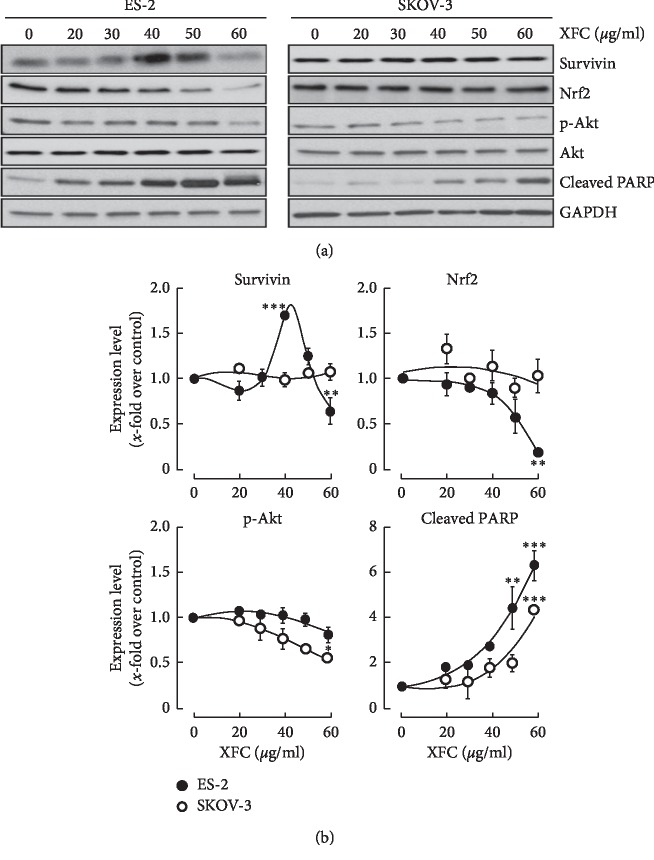
XFC extract triggers PARP cleavage in SKOV-3 ovarian cancer cells. Human ovarian clear cell carcinoma (ES-2) and human ovarian adenocarcinoma (SKOV-3) cells were cultured as described in Section 2. Treatment with various concentrations of XFC was performed in serum-free media for 24 hours. Cell lysates were harvested and then processed for (a) SDS-PAGE and Western blotting in order to assess the phosphorylation status of Akt, expression levels of Survivin, Nrf2, GAPDH, and cleaved PARP. (b) Levels of expression were quantified using scanning densitometry.

Different levels of alterations in apoptotic versus necrotic cell death balance as well as in cell cycling prompted us to investigate whether several intracellular stress biomarkers were also altered (Figure 6(a)). Interestingly, a transient increase in Survivin expression was observed in ES-2 cells whereas it remained unchanged in SKOV-3 cells, suggesting that the latter cell model did not require Survivin to resist the XFC stress induction. However, the global phosphorylation status of Akt decreased in ES-2 cells and even more significantly in SKOV-3 cells (Figure 6(b)). Expression of the nuclear factor erythroid 2-related factor 2 (Nrf2), an emerging regulator of cellular resistance to oxidants [24] believed to control the expression of antioxidant response element- (ARE-) dependent genes which regulate cellular resistance to oxidants [25], was found to decrease in XFC-treated ES-2 cells but not in SKOV-3 cells. This confirmed the chemoresistance phenotype of SKOV-3 cells. Finally, cleaved PARP immunoreactive material was significantly induced in ES-2 cells but was also induced in SKOV-3 cells. Altogether, this confirms the high drug resistance phenotype of SKOV-3 cells to cytotoxics as compared to the chemosensitive ES-2 cells. Nevertheless, our data still support some aspects of the cytotoxic properties of XFC that can circumvent this resistance phenotype.

### 3.6. XFC Alters Cell Cycle Regulators in SKOV-3 Ovarian Cancer Cells

**Figure 7:**
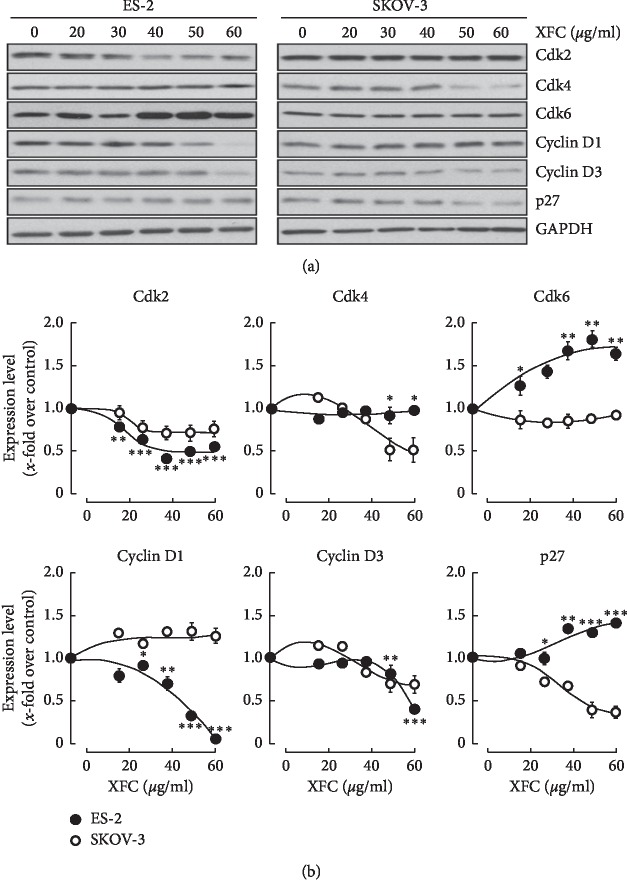
XFC extract inhibits CDK4 and cyclin D3 in SKOV-3 ovarian cancer cells. Human ovarian clear cell carcinoma (ES-2) and human ovarian adenocarcinoma (SKOV-3) cells were cultured as described in Section 2. Treatment with various concentrations of XFC was performed in serum-free media for 24 hours. Cell lysates were harvested and then processed for (a) SDS-PAGE and western blotting in order to assess the expression levels of Cdk2, CDk4, Cdk6, Cyclin D1, Cyclin D3, p27, and GAPDH. (b) Levels of expression were quantified using scanning densitometry.

A common mechanism of cell cycle arrest involves the upregulation of endogenous Cdk inhibitors, including p27^Kip1^ and p21, which prevent cell cycle progression by blocking Cdk activity [26]. Immunoblotting indicated that p27^Kip1^ levels did significantly increase in ES-2 cells (Figure 7(a)), whereas they significantly decreased in chemoresistant SKOV-3 cells (Figure 7(b)). When Cdk2, Cdk4, and Cdk6 expression levels were assessed, that of Cdk2 decreased significantly in ES-2 cells upon XFC treatment, whereas it was less altered in SKOV-3 cells (Figure 7(b)). Whereas Cdk6 only increased in ES-2 cells, Cdk4 in contrast decreased upon XFC treatment in SKOV-3 cells but remained unchanged in ES-2 cells (Figure 7(b)). Protein levels of Cyclin D1 in ES-2 cells were decreased drastically by XFC treatment, whereas they remained unchanged in SKOV-3 cells (Figure 7(b)). Cyclin D3 expression was significantly downregulated in both SKOV-3 and ES-2 cells.

### 3.7. SKOV-3 Ovarian Cancer Cells Cannot Reverse the Impact of XFC in a Wound-Healing Assay

**Figure 8:**
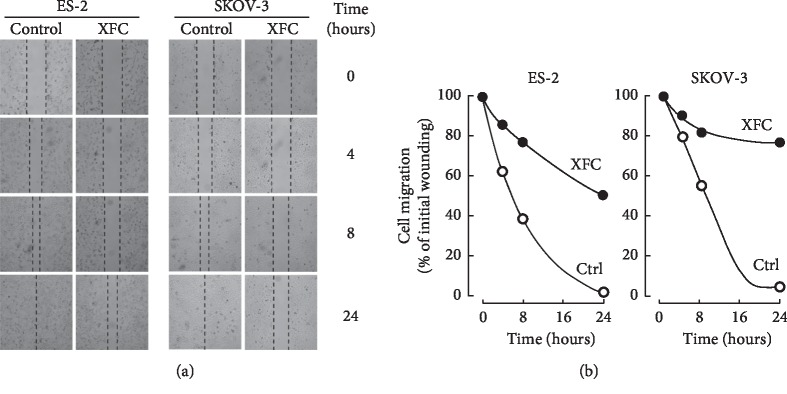
SKOV-3 ovarian cancer cells are resistant to the effect of XFC in a wound-healing assay. (a) Photomicrographs of ES-2 and SKOV-3 cell migration, in the presence or absence of 60 *μ*g/mL XFC, to the scratched zone at different time points (magnification, ×20). (b) Quantitative assessment of cells migrated into the scratched zone. For each condition, representative fields within the scratch were photographed. Data are representative of two independent experiments and are presented as a percentage of cell migration into the scratched zone in each culture medium group (control, 100%).

The effect of XFC on the ability of cells to migrate in response to a wound was next assessed (Figure 8(a)). While ES-2 cells were able to partly rescue wounding, XFC treatment in SKOV-3 cells prevented migration of the wound region (Figure 8(b)). This property suggests that XFC can halt SKOV-3 cell migration.

## 4. Discussion

Although ovarian cancer is the most deadly gynecologic malignancy worldwide, chemotherapy remains the mainstay treatment. Although the initial response to this treatment is generally promising, frequent recurrence in patients with advanced stages of the disease remains a therapeutic challenge. Thus, understanding the biology of chemoresistance is of great importance for overcoming this challenge and should benefit the survival of ovarian cancer patients. Although complex mechanisms underlie the development of ovarian cancer chemoresistance, here we show that XFC may significantly alter cell survival properties associated with this resistance phenotype in a cytotoxic, drug-resistant SKOV-3 ovarian cancer cell model. Importantly, SKOV-3 cells are documented to be resistant to tumor necrosis factor and to several cytotoxic drugs including diphtheria toxin, cis-platinum, and Adriamycin where efflux in drug accumulation was assessed *in vitro* against four platinum complexes, and where decreased drug accumulation reported to be in part responsible for acquired resistance to cisplatin [27].

In this study, it is demonstrated that XFC exhibited antiproliferative activity against several cell line models of breast, ovarian, and colon cancer. We found that the percentage of growth inhibition was dose-dependent with the best XFC antiproliferative effect against ovarian chemosensitive ES-2 and chemoresistant SKOV-3 cancer cells. While the two breast cancer cell and two colon cancer cell models tested herein were also found responsive to XFC treatment to some extent, their lower responsiveness may suggest that specific targeting and efficient pharmacological effects are conditioned by the differential molecular and cellular signature of a given cancer. This further prompts for future identification of the exact cell surface and/or intracellular biomarkers specifically involved in the actions of the active molecules within XFC. As such, it has been reported that XFC contains 3,4-dihydroxybenzaldehyde that inhibits human U937 cancer cells [28]. Two xanthanolide sesquiterpene lactones, 8-epi-xanthatin and 8-epi-xanthatin-5*β*-epoxide, have also been isolated from leaves and inhibit different cancer cell lines such as A549 (lung), SK-MEL-2 (melanoma), XF498 (CNS), and HCT-15 (colon) [13]. Whether these isolated molecules also alter ES-2 and SKOV-3 cell lines requires further examination although traditional medicinal research emphasises that the mixture of herbal extracts usually has lower toxicity and higher efficacy than individual molecules [29]. For instance, the crude extract of *Rabdosia rubescens* has shown higher synergistic effects at several concentrations than do its individual active ingredients. Furthermore, the total phenolic extract of blueberry was also found to significantly inhibit the growth of several oral (CAL27 and KB) and prostate (22RV1, RWPE-2, and RWPE-1) cancer cell lines [30]. As reported previously, the impact of XFC treatment is therefore not just related to one specific chemical compound, but it is the synergistic actions of different compounds that provide such an inhibitory effect [31]. According to the National Cancer Institute (NCI, USA) criteria, a crude extract would be considered as cytotoxic when an IC_50_ < 20 *μ*g/mL [32]. While the current IC_50_ (∼30–35 *μ*g/mL) of the XFC extract appears slightly higher, further fractionation will be required to identify the active component(s) that may alone or in synergy further represent promising candidates for anticancer drug R&D. However, such fractionation of XFC may result in the loss of its integrity and potential therapeutic potential. This further justifies the use of total extracts in the treatment of various diseases.

Natural or synthetic antimitotic molecules represent a considerable potential source for anticancer drugs because tumor cells are characterized by high mitotic activity as compared to normal cells. As such, antimitotic drugs have proven very effective against a wide range of tumors [33]. In response to induced mitotic spindle disturbances, cells are arrested in mitosis and, after prolonged mitotic arrest, undergo apoptosis, a feature of the anticancer effects elicited by these drugs [33–35]. Our study demonstrates that XFC selectively induces mitotic arrest in ES-2 and SKOV-3 cells, leading to decreased cell growth and viability in a dose- and time-dependent manner (Figure 4). XFC also inhibited normal mitotic progression by interfering with the metaphase to anaphase transition, consistent with previous data showing an antitubulin action of the extract [8, 17]. Our data, showing an impairment in anaphase entrance, demonstrates that XFC interferes with the normal function of the mitotic spindle, effects similar to those displayed by other well-known antimitotic drugs [36].

Recurrence and therapy resistance following chemotherapy recently highlighted an integral role of ovarian cancer stem cells (CSC) [37]. Interest has risen in overcoming the therapeutic resistance phenotype conferred by ovarian CSC through the use of chemotherapeutic drugs in combination with metabolism targeting approaches [38]. Whether XFC or dietary-mediated intervention can also target ovarian CSC death remains to be explored. Evidence suggests that deregulation of key pro- and antiapoptotic pathways represents a key event in the acquisition and maintenance of ovarian cancer chemoresistance [39]. Among these, Survivin was recently identified as an antagonist of chemotherapy-induced cell death in colorectal cancer cells [40]. Furthermore, the discovery of novel interactions between apoptosis and necrosis pathways confirms that chemoresistance may be multifactorial. In our study, we provide evidence that XFC is able to trigger apoptosis in both sensitive (ES-2) and chemoresistant (SKOV-3) ovarian cancer cells (Figures 2 and 3). Interestingly, significant XFC-mediated necrosis induction was only observed in SKOV-3 cells, suggesting that the ability of the ovarian cancer cell to escape the cytotoxic insult to XFC is a consequence of the overall necrotic balance response of that cell. Necrotic cell death mechanisms therefore appear to be preferentially involved in the chemoresistant SKOV-3 ovarian cell model (in contrast to ES-2 cells where most of the cell death mechanism rather involve apoptosis). This differential cellular effect, which correlates with the chemoresistance molecular signature of SKOV-3 cells, reinforces the concept that profound impact of XFC is required to induce cell death and that such characteristics may explain the time frame and versatile capacity of its cytotoxic molecules content. Tentatively, short-term (24 hours) induction of both necrosis and apoptosis is triggered by 400 *μ*g/mL XFC, and then switch from necrosis to late apoptosis completed upon long-term (48 hours) treatment.

Key proteins in cell cycle regulation, including c-myc, p21, Cdk4, and Cyclin D3, were all recently documented as potential prognostic factors in myoepithelial carcinoma of salivary glands [41]. Among the numerous target genes of MYC, Cdk4 and Cyclin D1 affect cell division since both regulate cell cycle progression and lead to enhanced proliferation [42]. Furthermore, Cdk inhibitors p15, p21, and p27 are downregulated by MYC, which impacts cell cycle progression in early and mid-G1 [42]. Our study highlights the capacity of XFC to specifically decrease Cdk4, Cyclin D3, and p27 expressions in chemoresistant SKOV-3 ovarian cancer cells (Figure 7(b)). This aspect is interesting as the formation of a ternary p27(kip1)/Cyclin D3/Cdk4 complex was recently described [43], and therapeutic targeting of the cyclin D3: CDK4/6 complex in T-cell leukemia envisioned as an efficient treatment for pediatric and adult T-cell leukemia where rapid cell cycle arrest in both mouse and human T-ALL cell lines was observed [44]. It is tempting to suggest that such similar targeting by XFC also takes place towards chemoresistant ovarian cancer cells. Moreover, XFC's ability to differentially target the microtubule dynamics and to further trigger necrosis events in that same model suggests that differential cell death decisions may be involved in line with the nature of the anticancer molecules content. The XFC-mediated mitotic arrest and increased necrosis that we observed in our SKOV-3 ovarian cancer cell model further appeared to be secondary to the accumulation of polymerized microtubules, in line with previously reported Taxol-induced transient mitotic arrest also associated with cell necrosis [45]. Finally, the caspase-independent apoptosis, triggered by the inhibition of cancer cell proliferation by berry juices, appeared similar to XFC treatments, to involve cell cycle arrest, as evidenced by downregulation in the expression of Cdk4, Cdk6, Cyclin D1, and Cyclin D3 in PC-3 prostate cancer cells [46]. Collectively, these data suggest that XFC contains anticancer molecules that could efficiently target crucial cell cycle division processes, which regulate chemoresistance. The closest antitumor impact and concentrations extrapolation one can perform at this point is provided from a recent study, which tested the *in vivo* antitumor impact of XFC to that of xanthatins fractionated from XFC in a colorectal xenograft model [47]. The authors elegantly demonstrated that daily administration for 15 days of 100 mg/kg XFC or of 5 mg/kg of xanthatins enriched fraction efficiently reduced tumor volumes to levels similar to that of a platinum chemotherapy drug at 6 mg/kg Oxaliplatin [47].

## 5. Conclusion

In conclusion, both of the cell death biochemical pathways believed to downregulate cell survival and the means by which XFC counteracts therapy resistance mechanisms still require more research. Our study provides the fundamental cellular mechanisms, which appear to be effectively targeted by XFC, and which may enable this treatment to circumvent some of the ovarian cancer cell chemoresistance mechanisms. Future perspectives should focus on identifying the exact active principles responsible for anticancer pharmacological activity. Furthermore, XFC impact on *in vivo* xenograft models may also be envisioned in small animal models and possibly administered in combination with current chemotherapy drugs.

## Data Availability

The data used to support the findings of this study are available from the corresponding author upon request.
